# Spices, herbs and allergic reactions in children: myth or reality — a narrative review with scoping elements

**DOI:** 10.3389/falgy.2025.1698559

**Published:** 2025-11-11

**Authors:** Elena Camelia Berghea, Gavriela Feketea, Maria-Teodora Cosoreanu, Olivia-Mihaela Popa, Marcela Daniela Ionescu, Corina Porr, Florian Berghea, Emilia Vassilopoulou

**Affiliations:** 1 Department of Pediatrics, Carol Davila University of Medicine and Pharmacy, 020021 Bucharest, Romania; 2 Marie Curie Emergency Children's Hospital, 041451 Bucharest, Romania; 3 Pediatric Allergy Outpatient Clinic, Pediatric Department, Karamandaneio Children's Hospital of Patra, Patra, Greece; 4 Department of Pathophysiology and Immunology, Carol Davila University of Medicine and Pharmacy, Bucharest, Romania; 5 Faculty of Medicine, Lucian Blaga University of Sibiu, Sibiu, Romania; 6 Allergology Department, County Emergency Clinical Hospital, Sibiu, Romania; 7 Carol Davila University of Medicine and Pharmacy, Bucharest, Romania; 8 Department of Nutritional Sciences and Dietetics, School of Health Sciences, International Hellenic University, Thessaloniki, Greece; 9 Pediatric Area, Fondazione IRCCS Ca' Granda-Ospedale Maggiore Policlinico, Milan, Italy; 10 Department of Clinical Sciences and Community Health, Università Degli Studi di Milano, Milan, Italy; 11 Department of Life Sciences, School of Life and Health Sciences, Universiy of Nicosia, Nicosia, Cyprus

**Keywords:** allergy, allergic reactions, spices, herb, mustard allergy

## Abstract

Spices and herbs are widely used for their flavor and therapeutic properties. This narrative review explores current evidence on spice and herb allergies in children, using a scoping approach to synthesize data from case reports, clinical, immunologic, molecular studies, regulatory sources, and previous reviews. Selected adult cases were included for context. Spice and herb allergies are increasingly recognized in children, with symptoms ranging from mild oral reactions to anaphylaxis. The most frequently implicated spices include mustard, celery, coriander, fennel, cumin, anise, pepper, and herbs from the Lamiaceae-family such as mint, oregano, and sage. Both IgE- and non-IgE-mediated mechanisms are involved, with cross-reactivity to pollens (birch and mugwort) being common. Diagnosis remains challenging due to limited standardized tests; oral food challenge is the gold standard. Management relies on strict allergen avoidance and emergency preparedness. Improved clinical awareness, diagnostic tools, and clearer labeling—especially regarding hidden allergens—are crucial for effective care.

## Highlights

- Spice and herb allergies are increasingly recognized in children, involving both IgE- and non-IgE-mediated mechanisms, with cross-reactivity being a common feature.
- Diagnosis is challenging due to the lack of standardized testing and the prevalence of hidden exposures. However, the oral food challenge remains the gold standard.
- Hidden exposures and inadequate labeling hinder effective avoidance, making strict allergen avoidance and emergency preparedness essential components of management.
- The most frequent implicated spices are mustard, celery, coriander, fennel, cumin, anise, pepper, and herbs from the Lamiaceae family such as mint, oregano, and sage.

## 1 Introduction

Spices and herbs have historically been integral to diet and medicine, valued for their flavor-enhancing properties, aromatic compounds, bioactive constituents, and potential therapeutic effects (1).

The use of spices and herbs dates to antiquity, an illustrative example being their common use in Ayurvedic and traditional Chinese medicine (2, 3). Curcumin, the active compound of turmeric, possesses antiallergic properties, determining histamine release inhibition (4). Black pepper showed the potential to ameliorate allergic inflammation (5). In the modern era, as a consequence of their increasing incorporation into various products (ranging from food to cosmetics) on one hand, and the presence of advancements in immunology and food science on the other hand, concerns were raised about the potential allergenic effects of these widely used ingredients (6, 7).

According to the International Organization for Standardization, herbs and spices are defined as “vegetable products or mixtures used for flavoring, seasoning and imparting aroma in foods” (8). They can be derived from various plant parts—including seeds, roots, flowers, bark, and leaves—and belong to diverse botanical families such as *Apiaceae* (coriander, cumin, fennel, celery, dill, anise, caraway), *Lamiaceae* (mint, oregano, sage, basil, thyme, rosemary), *Brassicaceae* (mustard, horseradish, wasabi), *Liliaceae* (garlic, onion, chives, shallots), *Solanaceae* (paprika, chili, bell pepper), *Zingiberaceae* (ginger, turmeric), *Piperaceae* (black and white pepper) (9, 10). These diverse botanical origins imply a rich allergenic potential due to shared and cross-reactive proteins, including profilins, lipid transfer proteins (LTPs), pathogenesis-related proteins (e.g., Bet v 1, Api g 1, Cor a 1), cross-reactive carbohydrate determinants (CCDs), and seed storage proteins, many of these cross-reacting with pollen allergens (6, 7, 11, 12).

Functionally, spices and herbs are categorized by their culinary use and the plant part they originate from. Spices generally refer to dried seeds, roots, bark, or fruits (e.g., cinnamon from bark, nutmeg from seed) (13). Herbs typically consist of the leafy green parts of non-woody plants (e.g., basil, parsley, mint). From an allergological standpoint, both groups can induce sensitization and elicit allergic reactions, although spices, due to their concentrated nature and presence in powders or blends, may pose a higher risk of inadvertent exposure and sensitization (6, 7, 14). Even though allergic reactions to spices and herbs are traditionally considered rare, emerging reports suggest they may be underdiagnosed and underestimated (6, 9, 15, 16).

### 1.1 Literature review strategy and methodology

This review follows a narrative format with scoping elements, aiming to map the existing literature and identify key themes, controversies, and gaps in knowledge related to spice and herb allergies.

A non-systematic, but structured search strategy was performed across PubMed, Scopus, and Web of Science using search terms such as “*spice allergy,” “herb allergy”, “spice hypersensitivity”, “spice anaphylaxis”, “food allergy in children,” “cross-reactivity spices,” “mustard allergy,”* “*celery-mugwort-spices syndrome”, and “hidden allergens”.* Case reports, clinical reviews, observational studies, immunologic studies, molecular studies, and regulatory documents were considered eligible. No time restrictions were applied; however, only articles written in English were eligible for inclusion. The initial search retrieved approximately 300 articles. Titles and abstracts were screened to exclude publications not directly addressing allergic reactions to spices, herbs, or their components (e.g., studies focusing on food flavoring chemistry or toxicology). After this initial screening, the full texts of about 100 articles deemed relevant were reviewed in detail. Additional studies were identified through manual searches of the reference lists of these papers to ensure comprehensive coverage of the available literature. Following application of the inclusion criteria—clinical relevance to spice allergy, immunological or epidemiological data, and clear diagnostic or mechanistic context—a total of 63 articles was selected and included in the final analysis.

## 2 Epidemiology

The true prevalence of spice and herb allergies remains uncertain due to inconsistent diagnostic criteria, lack of standardized testing extracts, and the frequent omission of spices in food labeling. The estimated prevalence in the general population ranges between 0.04% and 0.13% in adults (6, 10, 17). The percentage corresponding to spice allergy varied from 2% to 6.4% of the patients with food allergy (6, 10, 17). Data on children are sparse, but it was noted that, while spice allergy is considered uncommon, emerging pediatric case reports suggest that it may be more prevalent than previously thought (9, 15). In a Finnish study on parent reported food allergies, including 1,542 children, spice allergy was present in 0.4% of boys and 1.3% of girls (18). One of the most allergenic spices, mustard, is reported to be responsible for 1.1% of food allergies in children and up to 6%–7% of total food allergies (19, 20). When skin-prick tests were performed in patients with suspected food allergies to spices, the botanical family with the most frequent sensitization in both children (32%) and adults (23%) was *Apiaceae* (coriander, caraway, fennel, celery) (10). Ranking of the most frequent culprits indicates mustard, celery, coriander, fennel, cumin, anise, pepper, mint, oregano, and sage as leading triggers (21). Trends from allergy clinics in Europe suggest a gradual increase in spice-related reactions, particularly in association with pollen-food allergy syndrome (12). In Southern Europe, mustard and celery are predominant allergens, while in Northern Europe cross-reactivity with birch pollen increases the risk of celery, parsley, and coriander allergy. Other reports highlight fenugreek and chili peppers, reflecting regional diets or the import of Eastern culinary traditions (7, 22).

Regarding the gender distribution, women present an increased risk of developing this pathology, one of the causes being the presence of spices and herbs in the composition of several cosmetics (6, 14). Certain populations are also at increased risk of developing spice and herb allergies. Children with pollen allergies, especially to birch and mugwort, are more likely to develop cross-reactive hypersensitivity to spices like celery, parsley, coriander, and mustard (11, 12). Individuals with atopic dermatitis, particularly those with early onset, may show sensitization to spice and herbs allergens through impaired skin barriers (15).Patients with food-dependent exercise-induced anaphylaxis (FDEIA) may develop reactions to spices in specific contexts (11, 23).

The absence of comprehensive epidemiologic surveillance systems and the limited use of validated testing protocols for spices contribute to the underestimation of prevalence. Moreover, the wide use of non-standardized spice extracts for skin prick testing further complicates prevalence assessment (6, 16).

## 3 Routes of exposure

Particular for spice allergy is the variety of the exposure routes: oral ingestion, inhalation and skin contact (13, 24). Spices and herbs are ubiquitous in the European diet, being traditionally incorporated and more often through the globalization of cuisine. Moreover, they are present in non-food items such as toothpastes, mouthwashes, and lip balms (e.g., clove oil, mint), cosmetics and topical creams (e.g., turmeric, cinnamon, essential oils), herbal teas and infusions (e.g., sage, mint, fennel), aromatherapy and household products (6, 13, 24, 25). Both children and adults may be exposed to the non-dietary substances through dermal or mucosal contact, increasing the risk for sensitization via non-oral routes, particularly in the presence of atopic dermatitis or disrupted skin barrier function (13, 24). An additional concern is the adulteration or contamination of spice products. Studies have shown frequent instances of mislabeling or mixing with undeclared botanical substances, which may introduce novel allergens not expected by consumers (26, 27). Occupational exposure should also be taken into consideration, as there were reports of several cases such as contact dermatitis from workers handling cinnamon (28), work-related asthma in spice mill workers (29), and rhinitis to black pepper (30). Many allergic reactions are attributed not to a single spice, but to composite spice blends (e.g., curry powders, chili seasonings, herbal teas), making identification of the culprit allergen difficult (6, 9).

## 4 Etiology and pathophysiology

Adverse reactions to spices and herbs can occur through immunologic (allergic) and non-immunologic (irritative or intolerant) mechanisms, therefore distinguishing between these is essential for accurate diagnosis and management. The allergenic potential of spices is influenced by the extent of processing, as drying, grinding or cooking determine protein alterations that affect their capacity to elicit an allergic response (14, 31).

The most clearly defined mechanism involves IgE-mediated hypersensitivity, where exposure to spice allergens triggers an immediate allergic response. Sensitization ocurs through inhalation, oral ingestion and skin contact and upon re-exposure, IgE antibodies lead to the activation of specific immunological pathways with the development of symptoms such as urticaria, angioedema, bronchospasm, or even anaphylaxis (6, 9, 15).

A variety of spices, such as cinnamon, coriander, anise, fennel, cumin, mint and sage, may lead to allergic reactions that involve a non-IgE mediated mechanism, manifesting as delayed symptoms such as contact dermatitis, stomatitis, perioral dermatitis or gastrointestinal manifestations (6, 9, 31). These reactions are more difficult to diagnose due to the lack of reliable *in vitro* or *in vivo* tests (9).

A multitude of specific proteins have been identified and described as responsible for the allergic reactions to spices and herbs: pathogenesis-related proteins (Bet v 1, Api g 1, Cor a 1, Cum c 1 etc.), profilins (Api g 4, Cap a 2, Cor a 2 etc.), LTPs, CCDs, seed storage proteins (2S albumins), 7S-vicilin, defensins (6, 7, 32–34).

Cross-reactivity is a well-recognized immunologic phenomenon where IgE antibodies directed against one allergen also react with structurally similar proteins from unrelated sources (35). It represents a key mechanism by which sensitization to aeroallergens leads to reactions to botanically related foods (6). Several spice allergens are cross-reactive with pollen proteins due to shared epitopes such as profilins, LTPs, and pathogenesis-related proteins (PR-10) (11, 12). This forms the basis of pollen-food-allergy syndrome, which is especially relevant in patients with seasonal allergic rhinitis (36, 37). An important illustration of cross-reactivity is represented by the celery-birch-mugwort-spices syndrome, a complex pathology in which patients affected by respiratory sensitization to mugwort Artemisia develop food allergy to spices and vegetables like celery, carrot, parsley, fennel, and coriander due to shared epitopes (12, 34, 38, 39). Bet v 1 homologues and profilins are the main allergens responsible for this cross-reactivity (6, 34). Mustard-mugwort syndrome is another entity with clinical relevance, associating mugwort pollinosis and mustard hypersensitivity (11, 38).

**Figure 1:**
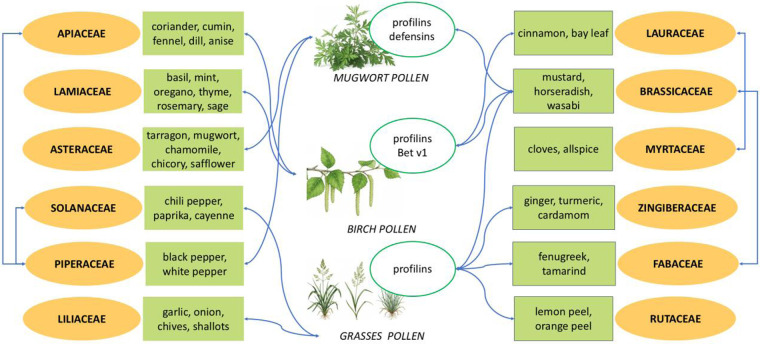
Patterns of cross-reactivity between pollen and spice allergens.

Figure 1 presents a schematic overview of known cross-reactivity patterns between spices, pollens, and related plant sources, emphasizing the role of similar allergens.

Spices from the same botanical family often share allergenic epitopes. Apiaceae family is characterized by a high intra-family cross-reactivity among coriander, fennel, celery, cumin, and dill (40). In the Lamiaceae family, sage, oregano, mint, thyme, and basil share cross-reactive components (7, 24). Mustard, horseradish, and wasabi, belonging to the Brassicaceae family, present high allergenic potential and shared epitopes (19).

Studies have also demonstrated significant overlap in sensitization to spices and other plant-derived foods. Figueroa et al. (2005) found that mustard-allergic patients also showed IgE reactivity to nuts (97.4%), legumes (94.7%), and Rosaceae fruits (89.5%) (11). Ivens et al. (2021) reported cross-reactive signals between chili peppers (Capsicum spp.) and Brazil nuts/hazelnuts in immunoassays—likely due to shared or structurally similar proteins (41). Cross-reactivity between fenugreek (a frequent component of curry) and peanuts was described by Faeste et al. (32), Takei et al. (42), and also by Che et al. (43), who also reported a possible cashew-sumac cross-reactivity leading to anaphylaxis. Dreskin et al. studied the importance of 2S albumins in the cross-reactivity between peanuts, tree nuts, and sesame seeds (44).

Some adverse responses to spices develop due to the inherent irritative properties of certain bioactive compounds. Capsaicin (found in chili) can cause burning, mucosal irritation, or bronchospasm; cinnamaldehyde (found in cinnamon), can provoke irritative contact dermatitis or oral burning; essential oils in herbs may induce dose-dependent irritant reactions (6, 7, 13). These effects are non-immune mediated, but can mimic allergic symptoms, complicating differential diagnosis.

## 5 Clinical manifestations

The clinical spectrum of allergic reactions to spices and herbs is broad, ranging from mild localized symptoms to severe systemic responses (Table 1). In children, the onset of allergic reactions can be early in life [a study by Rancé et al. (15) reported that the majority of children developed mustard allergy under the age of 3], but symptoms can also appear later on, in adolescence (16, 24).

**Table 1:** Clinical manifestations of allergic reactions to spices and herbs.

Type of reaction	Symptoms	Frequent determinant spices	Reference
Cutaneous manifestations	Urticaria Angioedema Contact dermatitis Oral allergy syndrome (OAS)	mustard, horseradish	Sharma et al. (19) Rancé et al. (15)
		pepper, garlic	Chen and Bahna (6) Lisiecka (7)
		oregano, mint, sage, rosemary, basil, thyme	Yazıcı et al. (24) Wagner et al. (12) Lisiecka (7) Benito et al., 1996 Miroddi et al.,2014
		caraway, cumin, anise	Lisiecka (7)
		chili, paprika	Ivens et al. (41)
		turmeric, ginger	Bradatan and Sabouraud (9) Liddle et al. 2006
		cinnamon	Mertens et al. (13) Ackerman et al. (28) Biron et al. (47) Calapai et al. (25) Isaac-Renton et al. (48)
Respiratory manifestations	Nasal congestion Rhinorrhea Sneezing Coughing Wheezing Bronchospasm	chili, paprika	Ivens et al. (41) Airaksinen et al. 2015
		garlic, onion, chives	Bradatan and Sabouraud (9) Van der Walt et al. (29)
		caraway, cumin, oregano, anise, rosemary	Lisiecka (7) Garcia-Gonzalez et al. 2002
		mustard	Rancé et al. (15)
		pepper	Perić et al. (30)
		mint	Szema and Barnett 2011
Digestive manifestations	Abdominal pain Vomiting Diarrhea Nausea	coriander, fennel, celery, caraway, cumin	Brussino et al. (40) Asero et al. 2021
		mustard	Rancé et al. (15) Figueroa et al. (11)
		turmeric, ginger	Bradatan and Sabouraud 2020
		pepper, oregano	Lisiecka (7)
		garlic	Hellu et al. (31)
Systemic manifestations	Anaphylaxis	mustard, horseradish	Sharma et al. (19) Rancé et al. (15) Figueroa et al. (11)
		coriander, fennel, celery, caraway, cumin	Brussino et al. (40) Worm et al. 2012 Lisiecka (7) Ebo et al. 2006 Unkle et al. 2012
		pepper, anise	Lisiecka (7) Gimenez and Zacharisen (51)
		fenugreek	Fæste et al. 2010 Che et al. (43) Joseph et al., 2018
		sage, mint, oregano	Yazici et al. (52) Lisiecka (7)
		dill	Chiu and Zacharisen 2000

Urticaria and angioedema are among the most common manifestations, particularly following ingestion or skin contact (15, 24). In a French study involving children with mustard allergy the most frequent manifestation was atopic dermatitis (51.8%), followed by urticaria and/or angioedema (37%) (15). Contact dermatitis may occur with topical exposure, such as through cosmetics, herbal teas, or in occupational environment (13, 28). More severe cases presenting as systemic contact dermatitis have also been reported (13, 45). The clinical effects of cross-reactivity in children with spice allergy are well illustrated by a case of a 13-year-old boy who developed angioedema after the separate ingestion of three members of the *Lamiaceae* family- oregano, sage, and mint (24). In the context of occupational exposure, one case of pustular allergic contact dermatitis was described as a reaction to aromatic herbs from *Lamiaceae* family (46). Moreover, in some cases of contact dermatitis localized or generalized recurrences can appear after eating the specific spices (6). Oral allergy syndrome (OAS)—including tingling, swelling of lips, tongue, or throat—can result from cross-reactivity with pollen allergens (12). Several cases of allergic contact stomatitis were reported to be induced by cinnamon from food or non-food products (25, 47, 48).

Nasal congestion, rhinorrhea, difficulty breathing, coughing, sneezing and wheezing may result from inhalation of airborne spice particles (particularly in food handling or occupational environments) or from ingestion of culprit foods (7, 29, 30, 49). One noteworthy case of immediate allergic reaction consisting of sneezing, severe nasal obstruction, rhinorrhea and conjunctivitis was reported in a 12-year-old patient after consuming saffron, this being the only known case of saffron allergy in children (16). Symptoms characteristic to allergic conjunctivitis like lacrimation and ocular itching can also appear after exposure to spices (7, 50).

Gastrointestinal symptoms such as nausea, abdominal pain, vomiting, or diarrhea may occur after ingestion of a multitude of spices from various botanical families, either through IgE-mediated mechanisms or delayed hypersensitivity (7, 15, 31).

Though uncommon, anaphylaxis has been documented in pediatric cases, most notably in reactions to mustard (15), coriander (40), black pepper (51), fenugreek (32, 43, 52), fennel, sage, mint and oregano (9, 24). A study from Germany reported that one of the most frequent food allergens that triggered anaphylaxis in adults was celery (53). Food-dependent exercise-induced anaphylaxis was associated with allergy to mustard and other cross-reactive foods (11) and was also described in a case of celery-mugwort-birch-spice syndrome (23).

## 6 Diagnostic approach

Diagnosis of spice and herb allergy is challenging because of hidden exposures, variable symptom patterns, and limited standardized tests. A structured diagnostic approach should integrate a thorough clinical history—the cornerstone of diagnosis—with appropriate testing and, when necessary, oral food challenges. Clinicians should assess the type, timing, and reproducibility of symptoms. Suspected spice/herb exposure must be extensively analyzed, including compound foods, cosmetics, or herbal teas (7, 13). Given the high frequency of hidden allergens, food diaries and recall of commercial food ingredients are essential (40, 54). Pollen sensitization history should be assessed, particularly to birch, mugwort, or grass, which may indicate cross-reactivity (11, 12). Another important aspect that must be evaluated is the association with exercise, infections, or medications, which may point toward FDEIA (11, 23). In cases of known food allergies, it is essential to consider the possibility of cross-contamination or the inclusion of allergens under unfamiliar names in prepared foods (55).

Skin Prick Testing (SPT) represents an important tool that may be useful in reaching the diagnosis, although it has some limits, such as the fact that standardized commercial extracts for spices are limited and often unavailable (6, 7, 15). Prick-to-prick testing with fresh spice/herb material is recommended when clinical suspicion is high and in cases of suspected cross-reactivity (24). Even with native spice extracts or dialyzed extracts (designed to remove irritants), correlation with clinical findings has shown limitations (56). However, positive skin prick or specific IgE tests may reflect sensitization rather than clinical allergy. Interpretation must consider possible cross-reactivity and irritant properties (6, 7, 57).

Serum-Specific IgE Testing are available for only a few spice allergens (e.g., mustard, anise, celery, oregano, thyme, black pepper, garlic) (6, 7, 15, 16, 31, 57). However, unlike SPT, serum sIgE is not influenced by skin irritants, providing a clearer picture of immune response. Positive results must be interpreted with caution due to limited sensitivity and specificity, but they may be useful in identifying cross-reactive patterns (1, 6, 15).

In the case of non-IgE-mediated reactions, patch testing may help identify delayed hypersensitivity (6, 13). These type of tests may be useful, but the optimal concentrations are difficult to estimate and the possibility of irritative reactions must not be excluded (6, 14, 58). Studies have shown variable results, with positive reactions to ginger, nutmeg, and oregano at certain concentrations (59).

Oral Food Challenge (OFC), represents the gold standard for diagnosis, involving controlled reintroduction of the suspected spice in titrated doses, and also using placebo samples, in a single or double blinded design (4, 10). Medical supervision is essential, especially for children with systemic reactions. In those with multiple plant food allergies or pollen sensitization, assessing cross-reactivity helps avoid misdiagnosis and unnecessary restrictions.

Emerging techniques, such as advanced multiplex immunoassays and immunoblotting, may help identify specific allergenic components and clarify cross-reactivity, though their application in spice allergy remains limited (29, 41, 60).

## 7 Management

Treatment strategies must therefore be individualized, based on confirmed allergy status, and aim to balance allergen avoidance with preservation of quality of life. Strict avoidance of the confirmed allergens remains the main management strategy (6). Once a spice or herb allergy is diagnosed via oral food challenge or compelling clinical evidence, families should be informed about cross-contamination risks and must be educated on reading ingredient labels and recognizing vague terms such as “spices,” “natural flavors,” or “aromatic herbs” that may conceal the allergen (6, 15, 54). Apart from food restrictions, patients must be aware of the possible non-dietary exposure, due to the presence of spice and herbs in the composition of various other products such as cosmetics, tooth paste, massage oil, fragrances, aromatherapy products, essential oils, alcoholic beverages, cleaning products (6).

In patients with a positive history for anaphylaxis or systemic reactions, emergency management is essential and must include prescription of epinephrine auto-injectors, written allergy action plans for home and school settings in children, training for caregivers and school personnel in recognizing symptoms and using emergency medications (53). Antihistamine medication proved its utility in ameliorating cutaneous and respiratory symptoms, while in more severe cases corticosteroids may be a treatment option during the most intense period of evolution (7).

Dietetic support is of high importance, particularly in cases requiring multiple food eliminations or when the allergen is part of cultural or daily diets. Improvements in food labeling policies are necessary for a more efficient management, especially for spices not currently included in mandatory allergen lists (61). In the meantime, families may need to contact manufacturers directly or prefer unblended, single-ingredient spices with transparent sourcing.

Currently, specific immunotherapy (SIT) is not considered an established therapeutic option for spice or herb allergies (62). In selected cases—particularly among individuals exhibiting cross-reactivity between food allergens and aeroallergens, such as in pollen-food allergy syndrome—immunotherapy directed against relevant inhalant allergens may confer partial clinical benefit (63). Further research is needed to clarify the potential role of allergen immunotherapy in this specific patient population.

## 8 Conclusion

Herbs and spices are widely used in food, health, and cosmetic products, yet their allergenic potential remains underrecognized. Although only a few botanicals—such as mustard, celery, coriander, cumin, fennel, and members of the Lamiaceae family— are clearly linked to IgE- or non-IgE-mediated reactions, these can range from mild symptoms to anaphylaxis, often due to cross-reactivity with pollens. In children, diagnosis is challenging because of hidden exposures, diverse sources, and lack of standardized tests. A personalized approach combining history, targeted testing, and oral challenges is essential, while management requires strict avoidance, education, and multidisciplinary support. Major gaps persist, including the absence of reliable pediatric epidemiological data, lack of standardized diagnostic extracts, limited understanding of non-IgE–mediated mechanisms, and incomplete labeling regulations, all of which hinder safe avoidance and public health strategies. Strengthened allergen labeling and surveillance systems are crucial to enhance patient safety and awareness.
